# A Rare Case of an Intracardial Ectopic Thyroid in the Right Ventricle

**DOI:** 10.3390/jcdd12020045

**Published:** 2025-01-26

**Authors:** Chuangwei Wei, Ying Zhao, Yanting Song, Dongting Liu, Nan Zhang, Jiayi Liu, Zhonghua Sun, Zhaoying Wen, Lei Xu

**Affiliations:** 1 Department of Radiology, Beijing Anzhen Hospital, Beijing Institute of Heart, Lung and Blood Vessel Disease, Capital Medical University, Beijing 100029, China; weichuangwei1205@163.com (C.W.); dongting0530@163.com (D.L.); ljy76519@163.com (J.L.); wenzhaoying11@163.com (Z.W.); leixu2001@hotmail.com (L.X.); 2 Echocardiography Medical Center, Beijing Anzhen Hospital, Beijing Institute of Heart, Lung and Blood Vessel Disease, Capital Medical University, Beijing 100029, China; yingzhaoecho@163.com; 3 Department of Pathology, Beijing Anzhen Hospital, Capital Medical University, Beijing 100029, China; 18611357782@sina.cn; 4 Discipline of Medical Radiation Science, Curtin Medical School, Curtin University, Perth 6102, Australia; 5 Curtin Medical Research Institute (CMRI), Curtin University, Perth 6102, Australia

**Keywords:** ectopic thyroid, cardiac ectopic thyroid, right ventricular mass

## Abstract

The ectopic thyroid gland is an abnormal development of the embryo. Most of the ectopic thyroid occurs in the path around the thyroglossal duct or on the lateral side of the neck. However, ectopic thyroid occurs in the heart, which is rare. We report a case of right ventricular ectopic thyroid. This case highlights the imaging characteristics of computed tomography (CT) and cardiac magnetic resonance (CMR) and discusses the underlying mechanisms for a timely diagnosis.

## 1. Introduction

Ectopic thyroid gland (ETG) refers to thyroid tissue not located in its normal anatomical position. The most common location is the base of the tongue, which accounts for 90% of the cases [1]. Furthermore, ETG may be also found in the mediastinum, larynx, trachea, esophagus, and cervical lymphatic system and even in subcutaneous tissues, such as the gallbladder, mesentery, and adrenal glands [2]. Intracardial ectopic thyroid is rare, and its prevalence remains unknown [3]. Among the various cardiac locations, the right ventricle is the most commonly observed site for ectopic thyroid tissue. Here, we report a case of right ventricular ectopic thyroid with computed tomography (CT) imaging and magnetic resonance imaging (MRI) features presented.

## 2. Case Report

**Figure 1:**
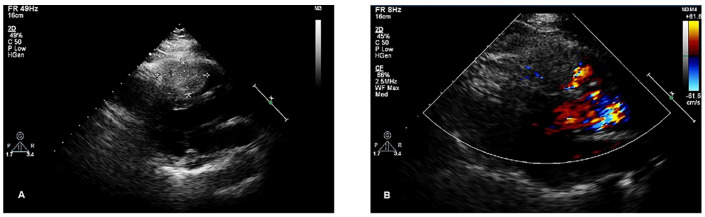
Transthoracic echocardiography of the lesion. (**A**) A 43 × 28 mm mass with medium echo appearance, neither mobile nor pediculated, adhering to the interventricular septum and extending to the right ventricular outflow tract. (**B**) Color Doppler flow imaging (CDFI): no significant blood flow signal was detected in the mass, and slight aliasing was noted in the right ventricular outflow tract.

A right ventricular mass was accidentally identified in a 56-year-old woman during chest CT for a respiratory infection at a local hospital. Transthoracic echocardiogram (TTE) revealed a cardiac mass, and the patient was admitted to our hospital for further diagnosis and treatment. The patient had a history of hypertension but no history of coronary artery disease. A grade 2/6 systolic murmur was heard between the second and third intercostal spaces at the left sternal border. The electrocardiogram showed sinus rhythm with possible right bundle branch block, and chest X-ray revealed a normal-sized heart. TTE indicated that the right ventricle was of normal size. A 43 × 28 mm mass with a moderate echo appearance, neither mobile nor pedunculated, was found to be adherent to the interventricular septum and extended to the right ventricular outflow tract. In addition, mild tricuspid regurgitation and normal mitral and aortic valves were noted (Figure 1).

**Figure 2:**
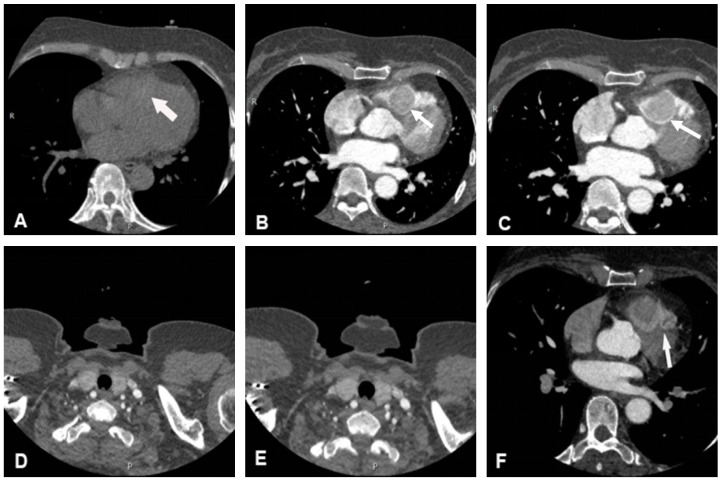
Cardiac CT imaging of the lesion. (**A**) The mass was slightly high in density on non-contrast CT scan. (**B**–**E**) The mass demonstrated obvious homogeneous enhancement on contrast-enhanced scan, which resembled the patient’s thyroid enhancement pattern. (**F**) CCTA images showed that the mass was perfused by a branch arising from the left anterior descending artery. White arrows indicate the location of the mass in each image.

Cardiac non-contrast CT confirmed the presence of a round mass slightly high in density within the right ventricle, with a CT value of 70 Hounsfield Units (HU). In the contrast-enhanced scan, early, rapid, and homogeneous enhancement was noted, with a CT value of approximately 131 HU, and further enhancement with a CT value of approximately 213 HU, which was similar in density to the patient’s thyroid gland on non-contrast and contrast-enhanced CT (Figure 2). Coronary computed tomography angiography (CCTA) suggested that the mass was perfused by a branch arising from the left anterior descending artery (Figure 2).

**Figure 3:**
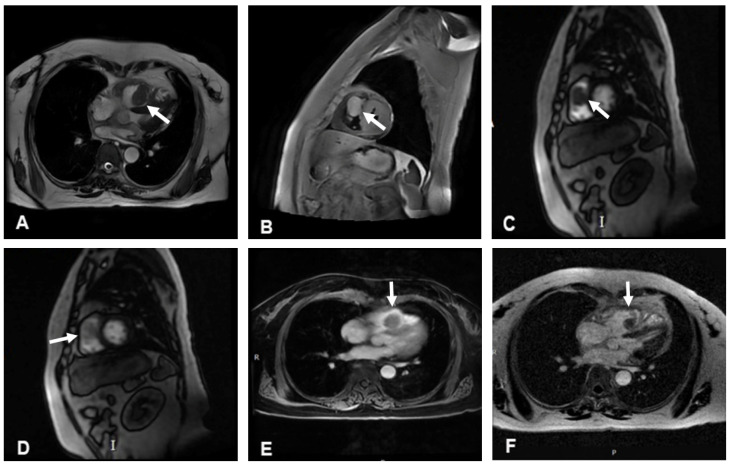
Cardiac magnetic resonance imaging. (**A**) Cine images of magnetic resonance imaging showed a hypointense mass. (**B**) The signal intensity of the mass was high on T2-weighted images. (**C**,**D**) The mass showed a rapid enhancement on CMR rest perfusion. (**E**) The signal intensity of the mass decreased rapidly on early gadolinium enhancement. (**F**) Myocardial delayed gadolinium enhancement was significantly lower than the blood pool signal at the same level. White arrows indicate the location of the mass in each image.

The cine cardiac magnetic resonance (CMR) images showed a hypointense mass with regular contours, which adhered to the interventricular septum and protruded into the right ventricular cavity. The signal intensity of the mass was high on T2-weighted images, without any signal decrease on the fat suppression sequence. However, pericardial or pleural effusion was not observed. CMR rest perfusion sequences demonstrated a rapid signal increase in the mass following an augmentation in the left ventricular blood pool signal, which implied that the mass was perfused by the aorta, and then a rapid decrease in signal intensity on early gadolinium enhancement (Figure 3). Moreover, myocardial delayed gadolinium enhancement was considerably lower than the blood pool signal at the same level.

**Figure 4:**
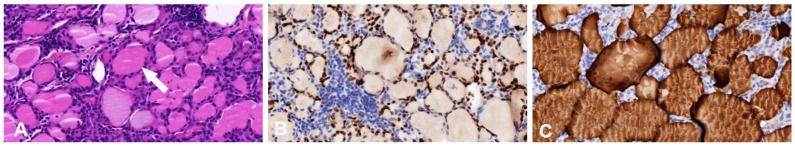
Postoperative pathology of the cardiac tumor. (**A**) H&E, ×40. The arrow shows thyroid follicles. (**B**) Thyroid transcription factor-1 (TTF-1), ×40. (**C**) Thyroglobulin (TG), ×40.

The patient had a structural abnormality of the heart that could not be treated with medications; thus, surgical intervention to remove the mass was recommended. Postsurgical chest X-ray and ultrasound examinations did not show any abnormality. Microscopic findings confirmed the absence of neoplastic cells in the mass, and only typical features of normal thyroid cells were observed. Immunohistochemical staining of the mass tissue revealed a positive reaction for thyroid transcription factor-1 and thyroglobulin (Figure 4). The final pathological diagnosis was intracardial ectopic thyroid.

## 3. Discussion

The incidence of primary cardiac autopsies is 0.05% [4], and approximately 80% of the cases are benign [5]. The incidence of metastatic tumors in the heart is approximately 1% [4], and intracardial ectopic thyroid is even rarer. This condition could be caused by the presence of thyroid rudiments dragged into the chest during the descent of the heart and great vessels during the early stages of organogenesis [6]. Intracardial ectopic thyroid affects mostly middle-aged women [7]. The most common cardiac sites are the right side of the ventricular septum and the right ventricular outflow tract [8,9], but it can also be found in the aorta [10,11,12,13] and left ventricular outflow tract [14,15].

The clinical manifestations of intracardial ectopic thyroid are nonspecific and often identified incidentally, sometimes presenting as dyspnea, chest pain, syncope, ventricular arrhythmia, or sudden death due to cardiac obstruction [14]. Patients with thyroid symptoms in the absence of localized thyroid disease or after thyroidectomy in the normal location should be comprehensively investigated for the possibility of ETG [16]. Most patients with intracardial ectopic thyroid exhibit normal thyroid hormone levels, which do not contribute to the diagnosis of the disease [17,18,19]. Intracardial ectopic thyroid has been shown to absorb iodine-123 on single-photon emission-computed tomography (SPECT) scans but not fluoride-18-labeled fluorodeoxyglucose uptake on positron emission tomography-CT (PET-CT) [16,20]. A study reported possible malignant transformation of cardiac ETG after thyroidectomy in the normal location, probably because increased thyroid-stimulating hormone following thyroidectomy could stimulate cardiac thyroid growth and even malignant transformation [21].

Intracardial ectopic thyroid is differentiated primarily from cardiac tumors and thrombus. Cardiac myxomas are attached by a stalk to the atrial septum in the oval fossa, and their range of movement is large. Contrast enhancement on CT often shows heterogeneous enhancement. Cardiac rhabdomyomas primarily affect infants and children [22]. On CMR cine images, rhabdomyomas appear to be isointense or slightly hyperintense on T1-weighted imaging and hyperintense on T2-weighted imaging, without significant enhancement after contrast medium administration. Cardiac hemangiomas have a broad base and no stalk. Contrast enhancement on CT shows centripetal filling from the edge to the center, with a time delay. The hemangioma appears to be hyperintense on T2-weighted imaging. Cardiac metastatic tumors arise as direct extensions or spread via hematogenous, lymphatic, or intracavitary routes of the primary tumor. Most common metastases to the heart originate from lung carcinoma (35–40%), followed by hematologic malignancies (10–20%) and breast cancer (10%) [23]. The most common location of metastasis is the pericardium, followed by the epicardium and myocardium. Ventricular thrombus typically occurs in the region of ventricular aneurysm or reduced ventricular motion.

Multimodal imaging is crucial in characterizing cardiac tumors. TTE is the most convenient imaging modality to determine the location of the mass, valve function, and outflow tract obstruction. Cardiac CT and CMR are beneficial in ascertaining the anatomic relationships among the mass, myocardium, and pericardium. In this case, the mass showed transient enhancement on CMR and obvious homogeneous enhancement on contrast-enhanced CT. This difference could be attributed to the different contrast agents used, with the mass absorbing the iodine contrast agent used in the CT contrast enhancement scan but not the gadolinium used in the CMR scan.

## 4. Conclusions

In summary, if a round, broad mass is found in the right ventricular outflow tract in a middle-aged woman, the imaging findings of the CMR enhancement scan are not consistent with those of the CT enhancement scan, and the mass is similar in density to that of the thyroid gland on non-contrast and contrast-enhanced CT, the possibility of intracardial ectopic thyroid should be considered after ruling out primary and secondary cardiac tumors.

## Data Availability

The data that support the findings of this study are not openly available due to reasons of sensitivity and are available from the corresponding author upon reasonable request.
